# Impacts of irrigated agriculture on food–energy–water–CO_2_ nexus across metacoupled systems

**DOI:** 10.1038/s41467-020-19520-3

**Published:** 2020-11-17

**Authors:** Zhenci Xu, Xiuzhi Chen, Jianguo Liu, Yu Zhang, Sophia Chau, Nishan Bhattarai, Ye Wang, Yingjie Li, Thomas Connor, Yunkai Li

**Affiliations:** 1 Center for Systems Integration and Sustainability, Department of Fisheries and Wildlife, Michigan State University, East Lansing, MI 48823 USA; 2 School for Environment and Sustainability, University of Michigan, Ann Arbor, MI 48109 USA; 3 College of Water Resources and Civil Engineering, China Agricultural University, Beijing, 100083 China; 4 Department of Biological and Agricultural Engineering, Texas A&M University, College Station, TX 77840 USA

**Keywords:** Environmental sciences, Socioeconomic scenarios, Sustainability

## Abstract

Irrigated agriculture has important implications for achieving the United Nations Sustainable Development Goals. However, there is a lack of systematic and quantitative analyses of its impacts on food–energy–water–CO_2_ nexus. Here we studied impacts of irrigated agriculture on food–energy–water–CO_2_ nexus across food sending systems (the North China Plain (NCP)), food receiving systems (the rest of China) and spillover systems (Hubei Province, affected by interactions between sending and receiving systems), using life cycle assessment, model scenarios, and the framework of metacoupling (socioeconomic-environmental interactions within and across borders). Results indicated that food supply from the NCP promoted food sustainability in the rest of China, but the NCP consumed over four times more water than its total annual renewable water, with large variations in food–energy–water–CO_2_ nexus across counties. Although Hubei Province was seldom directly involved in the food trade, it experienced substantial losses in water and land due to the construction of the South-to-North Water Transfer Project which aims to alleviate water shortages in the NCP. This study suggests the need to understand impacts of agriculture on food–energy–water–CO_2_ nexus in other parts of the world to achieve global sustainability.

## Abstract (web summary)

Local human activities can lead to cross-border environmental impacts through the food–energy–water–CO_2_ nexus. Here, the authors report wide variations in environmental impacts of irrigated agriculture across counties within the North China Plain under different environmental and socioeconomic scenarios.

## Introduction

Ensuring food security for a growing global population under resource constraints is one of the biggest global challenges^1^. Food production can also contribute to other global challenges, such as water scarcity, global warming, and pollution since food production consumes large amounts of water, energy and fertilizers and causes environmental burdens. Food, water, and energy provide a foundation for environmental and socioeconomic development. Global challenges, such as food insecurity, energy crises, water insecurity and global warming threatens sustainability in many regions worldwide^2–4^. The United Nations, therefore, recommended the 17 Sustainable Development Goals, such as achieving zero hunger, clean water, sustainable energy, and combatting climate change to transform our world^5,6^.

Food, energy, water, and CO_2_ emissions are highly interconnected, which is called a nexus relationship^7–13^. For example, water is used to produce food and energy (e.g., irrigation, hydropower, and bioenergy crop)^14^, and in turn, energy is required to pump and distribute water and produce food (e.g., water diversion projects, desalination, and irrigating)^15,16^. All of these processes generate CO_2_ emissions.

A growing body of research has explored the environmental impacts of food production and trade^16–21^. The environmental impacts of irrigated agriculture are particularly profound^22,23^ and the area of irrigated agriculture for domestic consumption and international trade is increasing globally^24,25^. However, there is a lack of systematic and quantitative analyses of irrigated agriculture’s impacts on food–energy–water–CO_2_ nexus across the agriculture and other areas simultaneously in the context of complex environmental and socioeconomic factors.

To address this important knowledge gap, we performed such analyses associated with food sustainability in China across food sending systems [the North China Plain (NCP), China’s major food production region with irrigated agriculture), food receiving systems (the rest of China) and spillover systems (Hubei Province, areas that are affected by interactions between sending and receiving systems) in the context of complex environmental and socioeconomic factors (e.g., climate change, diet change, irrigation technologies, crop planting strategies, and water diversion). The analyses were guided by the framework of metacoupling [environmental and socioeconomic interactions within and across borders^17^]. The metacoupling framework helps understand interrelationships among sending, receiving, and spillover systems that are connected by various flows (e.g., food trade and water transfer)^26–29^. It also suggests that the effects on spillover systems are largely ignored^30,31^, leaving an incomplete understanding of the environmental consequences of food production^2,21,30,32^. Assessing spillover effects can reveal the hidden environmental costs of food production and trade that can escalate local problems to national or even global catastrophes^33^.

China is the largest developing country in the world in terms of human population and faces many environmental challenges, including water scarcity, energy crisis, and intensive CO_2_ emissions under rapid population growth and economic development^34–37^. Ensuring food security while safeguarding the environment is, therefore, one of the greatest challenges for China and the rest of the world today. The NCP is China’s agricultural base and main producer of crops^38^, which provides approximate half of the national wheat and maize supply while consuming substantial water and energy and emitting CO_2_. Much of the food produced in the NCP is transferred to other regions throughout China. The NCP and other regions thus interact through the food trade between northern and southern China and food trade between central and western China^39,40^. Wheat and maize produced in the NCP take up to 95% of the agricultural land area in the region and comprise approximately 50% of China’s total wheat and maize production^38,41^. The government plans to apply water-conserving irrigation technologies in the NCP to alleviate water shortages and maintain crop yields^42^. To further reduce water pressure and support local industry and agriculture development in the NCP, the Chinese government implemented the South-to-North Water Transfer Project (SNWTP) to transport water from southern to northern China. The Middle Route has already been constructed. In this study, we focused on the Middle Route connecting Hubei Province and the NCP^43^. The SNWTP diverts water from Hubei Province (which receives little wheat and maize from the NCP^19^) to the NCP^44,45^. Assessing the relationships between environmental impacts related to food, water, energy, and CO_2_ emissions in different areas involved in food production, trade, and consumption can provide valuable information for managing sustainable food trade and environmental conservation across many regions of the world.

In this study, we addressed the following questions: (1) What crop production, energy footprint, water footprint, CO_2_ emissions, water and food sustainability are attributed to irrigated agriculture across the NCP? (2) What are the impacts of various environmental and socioeconomic factors (e.g., climate change, diet change, irrigation technologies, crop planting strategies, and water diversion) on the food–energy–water–CO_2_ (FEWC) nexus in the NCP? (3) What are the impacts of irrigated agriculture in the NCP on spillover systems (e.g., Hubei Province, Middle Route of the SNWTP). To answer these questions, we collected various kinds of data and employed life-cycle assessment. We also constructed 15 scenarios (Table 1) to simulate impacts of various factors on FEWC outcomes (Supplementary Table 1). We used the ratio of the total available water for agricultural use to the total water consumption of irrigated agriculture as an indicator for water sustainability (see details in “Methods”). The sustainable food supply indicator is set as the ratio of the actual amount of crop production to the sustainable amount of crop production for ensuring national food security in the rest of China (see details in “Methods”). Based on results from the analyses and simulations, we discuss the implications for solving potential environmental woes of resource consumption in the NCP (Supplementary Table 2). Results showed that food supply from the NCP enhanced food sustainability in the rest of China, but the NCP consumed over four times more water than its total annual renewable water. Under different scenarios with a combination of environmental and socioeconomic factors, the food–energy-water–CO_2_ nexus varied widely across counties in the NCP. Furthermore, the spillover systems also suffered large environmental impacts, such as land and water losses.

**Table 1:** Scenario settings for irrigated agriculture in the North China Plain.

Name	Scenarios	Settings
Baseline	Actual situation in 2010	Average temperature 13.1 °C, precipitation 454 mm, concentrations of CO_2_ 421.5 umol/mol, Double cropping system (winter wheat - summer maize rotation), Border irrigation, irrigated 4 times during growth period in normal flow years, 164.7 kg/y for grain intake, 9.5 billion m^3^/year water delivered through the Middle Route of the SNWTP
S1	Climate change for 2030	Based on Baseline, the average temperature changed to 14.3 °C, concentrations of CO_2_ changed to 491.5 umol/mol
S2	Reduced irrigation frequency in normal flow years	Based on S1, irrigated 2 times during growth period in normal flow years
S3	Rain-fed crops in high flow years	Based on S1, no irrigation occurred in high flow years
S4	Reduced irrigation frequency in low flow years	Based on S1, irrigated 3 times during growth period in low flow years
S5	Upgraded to drip irrigation	Based on S1, irrigation methods changed to drip irrigation
S6	Upgraded to sprinkler irrigation	Based on S1, irrigation methods changed to sprinkler irrigation
S7	Reduced irrigation and changed cropping system	Based on S2, cropping system changed to winter wheat - summer maize – spring maize
S8	Upgraded to drip irrigation and change cropping system	Based on S7, Irrigation methods changed to drip irrigation
S9	Upgraded to sprinkler irrigation and changed cropping system	Based on S7, Irrigation methods changed to sprinkler irrigation
S10	Diet change recommended by FAO A	Based on S1, 164.7 kg/y changed to 127.8 kg/y for grain intake
S11	Diet change recommended by FAO B	Based on S1, 164.7 kg/y changed to 94.0 kg/y for grain intake
S12	Diet change recommended by FAO C	Based on S1, 164.7 kg/y changed to 75.0 kg/y for grain intake
S13	Increased SNWTP water delivered	Based on S1, increased water delivered to 11.8 billion m^3^
S14	Increased SNWTP water delivered to maximum	Based on S1, increased water delivered to 14.1 billion m^3^
S15	Increased SNWTP water delivered to the maximum with diet changed and irrigation upgraded	Based on S8 + S12 + S14

## Results

### Environmental impacts in North China Plain

**Fig. 1:**
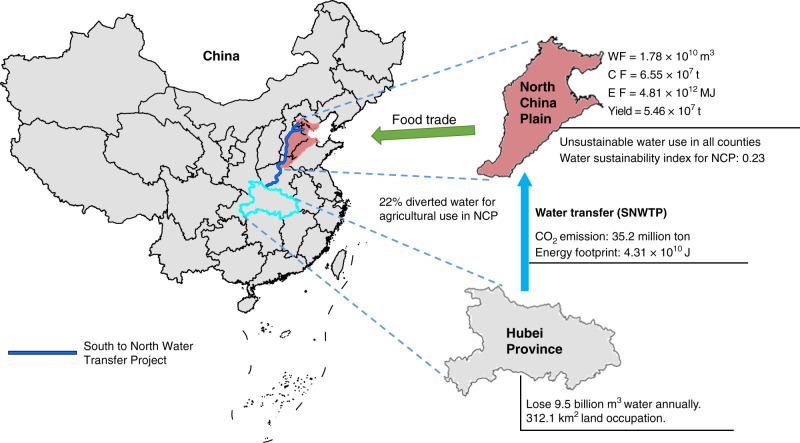
Metacoupling processes and associated environmental impacts. This Figure shows environmental impacts across the associated food sending system [North China Plain (NCP)], spillover system (Hubei Province), and receiving system (the rest of China). Note: An index value greater than 1 indicates sustainable food supply from the NCP or water use in the NCP. Only irrigation water use is considered in the NCP. The 9.5 billion m^3^/year water is the total water diverted by the South-to-North Water Transfer Project and 22% of the water went toward agricultural use in the NCP^89^. In other words, Hubei Province lost 9.5 billion m^3^ of water annually. The data for the base map was derived from the Resource and Environment Science and Data Center (http://www.resdc.cn/) which is publicly available. The boundary of the North China Plain and the route of the South-to-North Water Diversion Project were created by the authors (via ArcGIS version 10.1, ESRI).

**Fig. 2:**
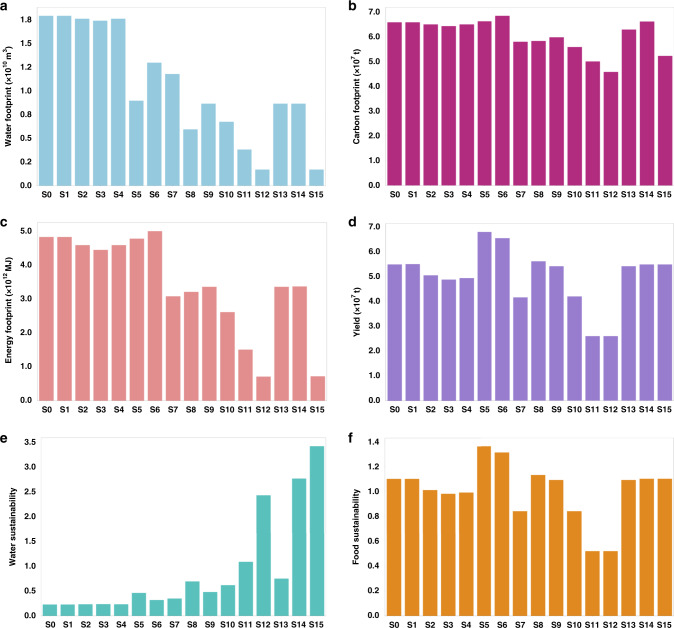
Environmental impacts in North China Plain under model scenarios. **a** Water footprint, **b** energy footprint, **c** carbon footprint, **d** crop yield, **e** water sustainability, and **f** food sustainability for irrigated agriculture in the North China Plain under scenarios S1–S15. Data are provided in the Source data file.

**Fig. 3:**
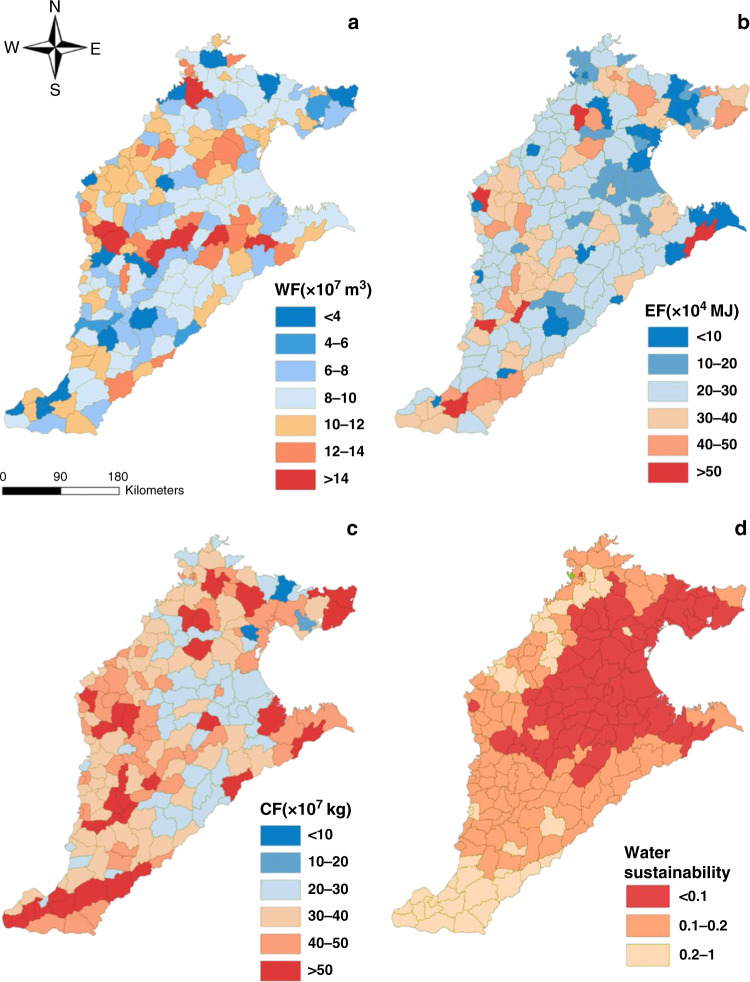
Spatial dynamics of environmental impacts in the North China Plain. **a** Water footprint, **b** energy footprint, **c** carbon footprint and **d** water sustainability in irrigated agriculture of the North China Plain. The data for the base map was derived from the Resource and Environment Science and Data Center (http://www.resdc.cn/) which is public available. The boundary of the North China Plain was created by the authors (via ArcGIS version 10.1, ESRI). Data are provided in the Source data file.

Surprisingly, all evaluated counties in the food sending system of the NCP had unsustainable water use due to irrigated agriculture (Figs. 1–3). However, crop production in the NCP fulfilled its share of responsibility for food sustainability in the rest of China. Total water consumption for irrigated agriculture in the NCP in 2010 was over four times the amount of renewable water available to the region for agriculture (inverse of water sustainability index, calculated as 1/0.23) (Supplementary Table 1). Irrigated agriculture in the NCP itself also consumed a large amount of energy and produced significant CO_2_ emissions (Figs. 1–3 and Supplementary Fig. 1; Supplementary Table 1).

### Environmental impacts under model scenarios

The water footprint, energy footprint, carbon footprint, crop yield, and water and sustainability indicators for the NCP varied widely under different scenarios (Fig. 2). The top three scenarios with the least water footprint (in descending order) include S11 (diet change B: 164.7 kg/y changed to 94.0 kg/y for grain intake), S15 (maximum water transfer plus diet change C), and S12 (diet change C: 164.7 kg/y changed to 75.0 kg/y for grain intake), while the bottom three (or the largest water footprint) include S1 (climate change), S2 (climate change plus reduced irrigation frequency in normal flow years), and S4 (climate change plus reduced irrigation frequency in low flow years), respectively (see details about the scenarios in Table 1). The top three scenarios for the least water footprints (i.e., S11, S15, and S12) were also ranked among the top three in terms of the lowest energy, and carbon footprints. S11 and S12, however, were among those with the lowest water and food sustainability. This is largely due to the lower crop yield associated with these scenarios (i.e., two of the bottom three lowest yield scenarios). The highest yield (and highest food sustainability) came from scenarios S5 (upgraded to drip irrigation), S6 (upgraded to sprinkler irrigation), and S8 (upgraded to drip irrigation and changed the cropping system). However, two of these three (i.e., S5 and S6) were among the ones with the highest energy and carbon footprints. These rankings of scenarios show that crop yield was driven by greater consumption of energy and water and not necessarily in a sustainable way. Overall, results indicate that water sustainability may not be guaranteed under 11 out of 15 scenarios [i.e., S1–10 and S13 (increased water delivered)]. Similarly, food sustainability may not be ensured under seven out of 15 scenarios [i.e., S2–4, S7 (reduced irrigation and changed cropping system), S10–12)]. Among the 15 scenarios, only two could achieve both water and food sustainability while at least water or food sustainability could not be achieved in the remaining scenarios.

There were large spatial variations in environmental consequences in the NCP (Fig. 3). Water sustainability appeared to have a decreasing pattern toward the south with the exception of few counties in the northwest. On the other hand, the water footprint, carbon footprint, and energy footprint show widespread heterogeneity across the counties. The variations in FEWC outcomes across counties seemed to be consistent with the heterogeneity in agricultural production, industrialization, farming practices, water utilization, among others. Across the 15 scenarios, there were also strong spatial differences in environmental impacts among counties of the NCP (Supplementary Figs. 2–5).

### Spillover effects

**Fig. 4:**
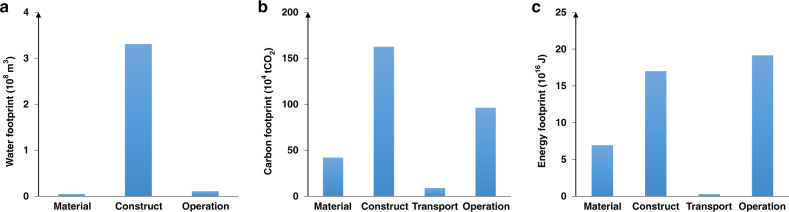
Water footprint, carbon footprint, and energy footprint at each stage of the South-to-North Water Transfer Project’s life cycle. **a** Water footprint; **b** carbon footprint; **c** energy footprint. Data are provided in the Source data file.

Spillover systems influenced by food trade absorbed substantial environmental burdens. For instance, Hubei Province, which is not directly involved in food trade with the NCP, lost significant amounts of land and water due to the construction and operation of the SNWTP (Fig. 1), which was installed partially to alleviate water scarcity in the NCP. Annually, the SNWTP diverts 9.5 billion m^3^ of water from Hubei Province to northern China, of which 2.1 billion m^3^ goes to agricultural use in the NCP. The SNWTP also occupies 310 km^2^ of land in Hubei Province. There were 149.3 km^2^ of cropland, 22.5 km^2^ of shrub land, 44.2 km^2^ of forest land, and 96.1 km^2^ of other land types such as grassland and barren land in Hubei Province being occupied by the construction of the SNWTP. CO_2_ emissions associated with the SNWTP were approximately 3.1 million tons (Fig. 4). Furthermore, there was substantial energy footprint throughout the life cycle of the SNWTP due to water transfer for food production in the NCP (Fig. 4).

## Discussion

This study provided the first assessment about how nexus trade-offs happened between different places under complex environmental and socioeconomic factors (e.g., climate change, diet change, irrigation technologies, crop planting strategies, and water diversion project.). Irrigated agriculture in the NCP largely influences China’s food security and related resource consumption and environment. We found although the spillover system (Hubei Province) did not participate in the interactions as a sending or receiving system, it was still affected and even suffered losses (e.g., water and land losses). Cross-sector woes also happened between different places, like how ensuring food security in the rest of China brought unsustainable water use, energy and carbon footprint in the NCP. In other words, the environmental impacts not only were interconnected between different sectors, but also interacted between different places across boundaries. Our findings revealed environmental stress across sending, receiving, and spillover systems due to irrigated agriculture in the NCP. The reasons may include China’s soaring crop consumption driven by its rapid economic development, rapidly growing population, and considerable declines in cultivated land area in southern China, as well as increases in crop production in northern China due to the development of agriculture and water conservation facilities in the region^46^. Water scarcity, energy consumption, CO_2_ emissions, and rapidly growing populations could interact to create complex socio-ecological challenges that, if left unaddressed, may threaten China’s sustainability.

The environmental impacts on the spillover system should be highlighted. Spillover systems are often overlooked in conventional studies and policymaking^31^. However, spillover systems can be affected by metacoupling, which can lead to severe environmental consequences^47^. Our study reveals how the food trade between the NCP and the rest of China places environmental burdens, including water loss and land occupation in Hubei Province, the spillover system.

Policies are needed to help address multiple aspects of environmental impacts in different areas simultaneously affected by the transfer of water. Deficit irrigation could be applied in areas that have yet to reach maximum water efficiency to minimize the trade-off between water sustainability in the NCP and food sustainability in the rest of China^48^. Since water sustainability is seriously threatened in the NCP and cannot support more food production in the long term, the crop production could be limited and be relocated.

More consumption-based policies (policies addressing consumption-related issues) could be combined with supply-oriented management to reduce pressures on food production and its associated environmental burdens and resource consumption. Applying supply-oriented management (management aiming at addressing supply-related issues) alone tends to increase water use, energy consumption, and CO_2_ emissions because it gives the false perception that natural resources and the ability of the oceans and atmosphere to absorb more CO_2_ emissions is limitless^49,50^. This false perception can encourage more intensive resource consumption and exacerbate environmental burdens associated with food production. Thus consumption-based policies such as those that encourage a diet shift to less resource-intensive crops and combat food waste in consumption side can help achieve sustainable development^51^. We suggest the Chinese government could consider both supply-side and consumption-side management simultaneously.

This study’s systematic assessment of environmental impacts across different systems associated with food trade and water transfers under various scenarios can be applicable to many other countries facing similar sustainability challenges. Such assessments are critical to achieving sustainable development since global food trade has proliferated in response to global challenges (e.g., water scarcity, food insecurity, CO_2_ emission, and energy crisis)^2,21,32,52,53^. In this study, the integrated framework of metacoupling helped fill important knowledge gaps through a comprehensive assessment of environmental impacts associated with food trade and water transfers across multiple systems. Due to data limitations, however, we cannot conduct analyses at the household level. Also, although current scenarios have considered various conditions, they still may not completely represent complex environmental and socioeconomic interactions in irrigated agriculture. Thus, future research could investigate the solution space more broadly and comprehensively to understand adaptation strategies by considering factors such as population growth, household dynamics, government investment and technology improvement, and natural disasters. For example, impacts of other technologies like gravity-based irrigation systems can be assessed to see if they perform better than other technologies for irrigated agriculture. Also, other socioeconomic factors about irrigated agriculture, such as robust water accounting and measurements, and the incentives and behavior of irrigators to subsidies could be included and assessed to avoid unexpected consequences^54^. Further research is also needed to go beyond the focus on environmental impacts by including socioeconomic impacts, such as poverty, social equality, health, and well-being. Such research can present a more comprehensive assessment of the socio-ecological effects associated with food trade and water transfers to help achieve global sustainable development.

## Methods

### Data sources

We obtained agrometeorological data from 1986 to 2010 from the Meteorological Data Sharing Service System of National Meteorological Information Center of China, and basic agricultural data (e.g., cultivated area, nitrogen use, winter wheat and summer maize production, and areal extent of other crops) in the NCP at the county level from the Agricultural Information Institute of Chinese Academy of Agricultural Sciences. We obtained crop evapotranspiration measurements for summer maize and winter wheat from Luancheng Agro-Eco-Experimental Station of the Chinese Academy of Sciences. We compiled data about SNWTP’s construction materials, work items and quantity of work from the Feasibility Study Report of South-to-North Water Transfer Project—Middle Route. This study focused on the Middle Route because it transfers water from Hubei Province to the NCP. The CO_2_ emission factors and energy intensity factors of each material used for construction were derived from ELCD (European Life Cycle Database), IPCC (Intergovernmental Panel on Climate Change) and Ecoinvent databases^55^. According to the general report of the SNWTP, all construction processes would follow the Hydraulic Construction Mechanical Quota 2002, selecting machines and determining machines’ work hours. The hydraulic quota 2002 was used to define the energy consumption of each unit of the work process.

### Metacoupled systems

We applied the metacoupling framework^17^ to define the systems in the scope of this study and identify the ways in which the systems were interconnected through food trade and water transfer. The NCP and regions throughout the rest of China are coupled human and natural systems, with human-nature interactions within each (e.g., crop production, energy and water consumption and CO_2_ emissions in the NCP; food consumption in the rest of China; water loss, land use, energy consumption and CO_2_ emissions in spillover systems), connected through the flows of food and water. The NCP is the sending system of food, and the regions throughout China that are supported by food from the NCP constitute the receiving system. Hubei Province is the spillover system because although it is not directly involved in food trade with the NCP^19^, it is the source site of the SNWTP, which diverts water from Hubei Province to the NCP for crop production there.

#### Life cycle assessment (LCA) of the chains of irrigated agricultural production

The use of LCA, performed according to ISO14040 & ISO14067^56^, is an established technique used for years to assess environmental impacts associated with a product throughout its life cycle^57^. Within the LCA model all impacts (e.g., water use, energy footprint and CO_2_ emissions, etc.) are measured by functional unit and are thus connected at the time of consumption to that unit^58^. LCA can be a viable technique for determining environmental thresholds. In this study, LCA and footprint methods were coupled to take advantage of their strengths and complementarities. Water, energy and carbon footprints were calculated through the LCA method (Supplementary Fig. 6). As for water footprint, the green, blue and grey water footprint was considered.

For both LCA and footprints, the analyses focused on five stages of agricultural operations during the life cycle: tillage, sow, irrigation, fertilization, and harvest (Supplementary Fig. 7). For both footprints and LCA the quantitative description of the inputs and outputs to/from the studied system is required. Irrigation stage includes irrigation energy consumption, irrigation equipment input, etc. The fertilization stage includes fertilizer inputs and fertilization equipment. The labors input and machinery depreciation occur at all five stages. WF accounting requires the inventory of the inputs and outputs in terms of water flows (blue, green, and grey as stated above). LCA requires the inventory of all the types of material inputs (mainly natural biotic and abiotic resources, including water) and outputs (mainly emissions in gaseous, solid and liquid forms, the latter related to water pollution and, hence to grey water) and the entering and exiting energy flows.

### Water footprint from crop production

#### WF_*cons*_

*WF*_cons_ of crop production is the total actual consumption of water within its whole production chain. Often, it is difficult to directly measure *WF*_cons_ and thus the indirect water requirement method is used. The crop water requirement is assumed as the water via crop evapotranspiration under optimal conditions, which is calculated by multiplying the reference crop evapotranspiration with a crop coefficient. Because actual crop growth is not always in optimal conditions, actual evapotranspiration should be less than optimal crop evapotranspiration and a water stress coefficient is introduced. The main factors that affected crop evapotranspiration include precipitation, air temperature, pressure, sunshine hours, wind speed, crop type, soil condition, and planted time. The calculation functions are given below^59,60^.

1 $$WF_{{\mathrm{cons}}} = \frac{{ET_{\mathrm{a}} \times A \times B}}{Y}$$

2 $$ET_{\mathrm{a}} = K_{\mathrm{s}} \times K_{\mathrm{c}} \times ET_0$$

3 $$ET_0 = \frac{{0.408\delta \left( {R_{\mathrm{n}} - G} \right) + \gamma \frac{{900}}{{T + 273}}U_2(e_{\mathrm{s}} - e_{\mathrm{a}})}}{{\delta + \gamma (1 + 0.34U_2)}}$$

Where *ET*_a_ (mm) is the actual crop evapotranspiration; *A* (km^2^) is the total plantation area; *Y* (kg) is the total crop yield; *B* is the unit conversion constant, equaling to 1000 here; *K*_s_ is water stress coefficient and is often assumed to be 1 for water requirement method; *K*_c_ is crop coefficient comparing to reference crop evapotranspiration; *ET*_0_ (mm) is reference crop evapotranspiration; *R*_n_ (MJ m^−2^ d^−1^) is net radiation on crop surface; *G* (MJ m^−2^ d^−1^) is soil heat flux; T (°C) is average air temperature; *U*_2_ (m s^−1^) is wind speed at 2 meters aboveground; *e*_s_ (kPa) is saturation vapor pressure; *e*_a_ (kPa) is measured vapor pressure; δ (kPa °C^−1^) is the slope of the curve between saturation vapor pressure and temperature; *γ* (kPa °C^−1^) is hygrometer constant.

For our proposed water consumption method, the actual crop evapotranspiration in Eq. (1) is calculated from the crop water production function (CWPF) shown below.

4 $$y = aET_{\mathrm{a}}^2 + bET_{\mathrm{a}} + c$$

5 $$ET_{\mathrm{a}} = {\mathrm{min}}\left( - \frac{b}{{2a}} \pm \sqrt {\frac{y}{a} + \frac{{b^2}}{{4a^2}} - \frac{c}{a}} \right)$$

Where, *a, b, c* are regression coefficients; *y* (kg/ha) is unit area crop yield.

#### WF_*blue*_ and WF_*green*_

*WF*_blue_ is the volume of consumed surface water and groundwater for producing goods or delivering services. *WF*_green_ is the volume of consumed rainwater during the production process. This is particularly relevant for agricultural and forestry products, including the total rainwater evapotranspiration (from fields and plantations) plus the water incorporated into the harvested products^61^. The blue and green *WF* can be calculated by the following equations:

6 $$WF_{{\mathrm{cons}}} = WF_{{\mathrm{blue}}} + WF_{{\mathrm{green}}}$$

7 $$WF_{{\mathrm{blue}}} = \frac{{ET_{{\mathrm{blue}}} \times A \times B}}{Y}$$

8 $$ET_{{\mathrm{blue}}} = \max (0,ET_{\mathrm{a}} - P_{{\mathrm{eff}}})$$

9 $$WF_{{\mathrm{green}}} = \frac{{ET_{{\mathrm{green}}} \times A \times B}}{Y}$$

10 $$ET_{{\mathrm{green}}} = \min (ET_{\mathrm{a}},P_{{\mathrm{eff}}})$$

11 $$P_{{\mathrm{eff}}} = \sigma P$$

Where *ET*_blue_ (mm) and *ET*_green_ (mm) are evapotranspiration of blue and green water, respectively; *P*_eff_ (mm) and *P* (mm) are effective rainfall and total rainfall within crop growth period, respectively; *σ* is the effective utilization coefficient of rainfall.

#### Crop water production function

Crop water production function (CWPF) is the mathematical expression that describes the relationship between water use and crop production for a certain kind of crop. The function is mainly influenced by sunshine and heat factors such as photosynthetically active radiation and effective accumulated temperature, and agricultural production factors such as soil organic matter, crop types and varieties^62^. In the North China Plain (NCP), annual average photosynthetically active radiation ranges from 2290 to 2524 MJ m^−2^. The effective accumulated temperature (EAT) of winter wheat and summer maize during their whole growth periods are 1298–2605 °C and 2077–2413 °C, respectively. Soil organic matter in 82.9% of the area is between 0.5% and 1%. These numbers suggest that for winter wheat there is only large spatial variation in its EAT while little variation in other production factors. Thus, we divided the total plantation area of winter wheat into three zones based on the EAT and used different CWPFs to improve the estimation accuracy. Because the spatial distribution of EAT did not exactly match the administrative boundaries of counties, the EAT for the majority of counties’ land area was used. For summer maize, given there is little variation for all production factors affecting the CWPF, the same CWPF was used for all counties for a certain year.

Meanwhile, the CWPF may vary in a long period due to changes in crop variety (e.g., drought resistance, unit area yield). In addition, data of CWPF may not be available every year. For this study, for winter wheat, we obtained CWPF data in years 1986^63^ and 2007^64^ for zone 1, in years 1990^65^ and 2003^61^ for zone 2, and in years 1986^63^ and 2008^66^ for zone 3, respectively. For summer maize, we acquired CWPF data in years 1984^63^, 2000^67^, and 2008^66^. Based on the obtained CWPF data in each two-consecutive time point, we then estimated the corresponding CWPF for each year with the assumption that the increment of crop yield per unit volume water consumption is equal across years.

In order to test the accuracy of the two methods, we took the measured data^61^ of ETc (from Luancheng Agro-Eco-Experimental Station of the Chinese Academy of Sciences, which is located in the northern part of the NCP.) to compare with data of ETc calculated from CWPF method and water requirement method. We mapped a scatter diagram with a *y*-axis of CWPF method and water requirement method, an *x*-axis of the measured data of ETc. The measured data are from 1986 to 2009 for summer maize and 1986 to 2007 for winter wheat. For both methods, the more discrete degree of the points, the less accuracy of the method.

#### WF_*grey*_

The *WF*_grey_ is an indicator of freshwater pollution that is associated with a product over its full production chain. It is calculated as the volume of water required to dilute pollutants to meet water quality standards. The *WF*_grey_ can also be divided into surface and ground sources.

We focused on the *WF*_grey_ of nitrogen because it was intensively used in the NCP and potentially had the most severe pollution since it can easily be transported in soil, surface water, and groundwater. Soil phosphorus often easily generates chemical reactions with other soil minerals and produces chemical compounds that are not readily soluble, resulting in less pollution. Potassium ions can be easily attracted by soil colloids and are not easily filtered. Therefore, the pollution from phosphorus and potassium fertilizers can be ignored when assessing *WF*_grey_. For chemical pesticides, given there were various types of used chemical pesticides with distinct maximum allowable concentrations, it was very difficult to collect the data. Thus, we did not take them into account.

The nitrogen application amount in the NCP had an average amount of 433.38 kg ha^−1^ and a range from 179.76 (min) kg ha^−1^ to 879.11 (max) kg ha^−1^. The spatial distributions of winter wheat and summer maize are similar; however, on average the nitrogen application amount of winter wheat (223.56_average kg ha^−1^) is higher than that of summer maize (212.43_average kg ha^−1^). The calculation functions for grey WF are shown below.

12 $$WF_{{\mathrm{grey}}} = \frac{{(\alpha _{{\mathrm{total}}} \times AR){\mathrm{/}}(C_{{\mathrm{max}}} - C_{{\mathrm{nat}}})}}{y}$$

Where *α*_total_ is total leaching fraction, measured to be 25.0%^68^; AR (kg ha^−1^) is per hectare application amount of chemical fertilizer; *C*_max_ (g L^−1^) and *C*_nat_ (g L^−1^) are the maximum acceptable concentration and natural concentration of the chemical fertilizer, respectively.

#### Sustainability of water use

The sustainability of grain production from a WF perspective can be reflected through the use intensity of total available water for agricultural use (*I*_total_). The higher the use intensity, the less sustainable for grain production. The water sustainability indicators can be calculated as follows.

13 $$I_{{\mathrm{total}}} = WR_{{\mathrm{agri}},{\mathrm{total}}}{\mathrm{/}}WF_{{\mathrm{total}}}$$

Where *I*_total_ is the sustainability indicator of total available water for agricultural use; *WF*_total_ is total WF for winter wheat and summer maize here; *WR*_agri,total_ is total water resources for agricultural use^69^. For *WF*_total_, the average values from 1986 to 2010 were calculated.

### Water sustainability

To assess the sustainability of water use involved in crop production in all NCP counties from the perspective of water quantity, we used the ratio of the total available water for agricultural use to the total water consumption of irrigated agriculture production (total direct and indirect consumption of water in the whole production chain) as an indicator for water sustainability (see Supplementary information for further descriptions and details on calculation method)^70,71^. An indicator value greater than 1 represents sustainable water consumption. The greater the value of the indicator, the greater the sustainability of water consumption associated with crop production. We then mapped the spatial distribution of water sustainability across all NCP counties using ArcGIS software (Esri Inc., Redlands, CA).

Because the actual amount of water consumed by crop production might differ from the amount calculated from conventional methods that estimate the required amount, we developed and used an alternative method of calculating water consumption based on the crop water production function and compared our results with those of conventional methods. We found the estimate from the conventional water requirement methods was significantly higher than actual measurements at the Luancheng monitoring station and our method showed higher accuracy. After confirming the greater accuracy of our methods compared to conventional methods, we applied our method to calculate the water consumption of crop production. All statistical analyses were performed using Stata 13.

### CO_2_ emissions and energy footprint from crop production

The process of agricultural production has large amounts of CO_2_ emissions. We calculated the CO_2_ emissions from irrigated agriculture (*CF*) using Eq. (14):

14 $$CF = CF_{\mathrm{F}} + CF_{\mathrm{I}} + CF_{\mathrm{M}} + CF_{\mathrm{H}} + CF_{\mathrm{S}} + CF_{\mathrm{P}}$$

Where *CF*_F_, *CF*_I_, *CF*_M_, *CF*_H_, *CF*_S_ and *CF*_P_ represent CO_2_ emissions from fertilizer application, irrigation, mechanical work, labor input, seed input, and pesticide and herbicide use, respectively.

We calculated the energy consumed through the whole crop production chain *E*_1_ through the following Eq. (15):

15 $$E_1={\it{E}}_{\rm{{W}}} + E_{\rm{M}} + E_{\rm{L}} + E_{\rm{F}} + E_{\rm{P}} + E_{\rm{I}}$$

Where *E*_W_*, E*_M_*, E*_L_*, E*_F_*, E*_P_ and *E*_I_ represent energy input for seeds, machine use, labor, fertilizer, pesticides, and irrigation, respectively.

### Simulation scenarios

We set the scenarios in Table 1 to simulate impacts of various factors on FEWC outcomes.

### Scenarios and crop production patterns

The assessment of water, energy and nitrogen footprint of irrigated agricultural production and production processes in the North China Plain under different scenarios considered climate change, irrigation systems and methods, dietary structure adjustment, water transfer through the South to North Water Transfer Project. The results in this section reflect spatial differences at the county-level calculations to analyze geospatial features^72^. The specific settings are as follows:

### Climate changes

We considered scenarios with different levels of climate change^73,74^. This paper simulated different climate scenarios and considerd three factors: precipitation (average water year), temperature, and carbon dioxide concentration. The reference crop transpiration ET0 was calculated by the Penman-Montieth formula. According to the research conclusion of ref. ^75^, the climatic factors were all calculated according to the trend from 2010 to 2030. This paper used the AquaCrop model to predict crop yield. The input modules mainly included crops, weather, soil types, groundwater, field management, and initial conditions. Technical model was based on the current technical model (mechanical tiller planting machine harvesting, ground furrow irrigation, irrigation 4 times under normal water years, surface water of irrigation water source: groundwater = 1:9, planting density 20 kg/mu, fertilization converted to pure N, P, K = 30, 10, 5 kg/mu, “Guiding Opinions on Scientific Fertilization Technology for Winter Wheat in North China Plain”)^76,77^.

### Agricultural production patterns

The agricultural production model mainly considers three categories: reducing irrigation volume, changing the rotation system, and upgrading irrigation methods. In order to be close to the actual production situation, this paper estimated the output under different irrigation and precipitation conditions through the Aqua-Crop model. Specifically, this paper included the reduction of irrigation to 2 times in normal water years, to 0 times (rain-fed) in high water years, and to 3 times in low water years. According to the Comprehensive Action Plan for Groundwater Overdraft in North China, the plan for water-saving irrigation area in the North China Plain is 58.62 million hm^2^ in 2020^60^. This paper evaluated the output and water, energy and carbon footprint when sprinkler irrigation and drip irrigation were extended to 58.62 million hm^2^ (Supplementary Table 3). The extension area was allocated to the counties according to the proportion of the existing cultivated land area.

#### Water transfer engineering and diet changes

From the perspective of consumption, this paper looked at the output demand under dietary changes, calculated the required output backwards, combined the production technology model of the first part of the scenario analysis, calculated the corresponding water energy carbon footprint, and explored the sustainable production model under dietary adjustment^78,79^.

### Sustainable food supply

We set an indicator of sustainable food supply to assess the impacts of crop production in the NCP on food security for the rest of China^80,81^. According to the Major Consulting Project of Chinese Academy of Engineering “China’s Agricultural Water Demand and High Efficient Farming Construction of Water Saving”^82,83^, in 2010, a wheat yield of 5,895 kg/hm^2^ and maize yield of 5,835 kg/hm^2^ in the NCP were required to fulfill its share of responsibility for the national food security. This project assigned part of the food production task to the NCP, which is partially responsible for ensuring national food security. We, therefore, calculated the sustainable food supply indicator as the ratio of the actual amount of crop production to the sustainable amount of crop production which is derived from multiplying the crop yield requirements with their cropland area. An indicator value greater than 1 indicates that sustainable food supply is achieved. A higher value in the sustainable food supply indicator indicates greater sustainable food supply for the rest of China.

### Food–energy–water–CO_2_ nexus in the NCP

**Fig. 5:**
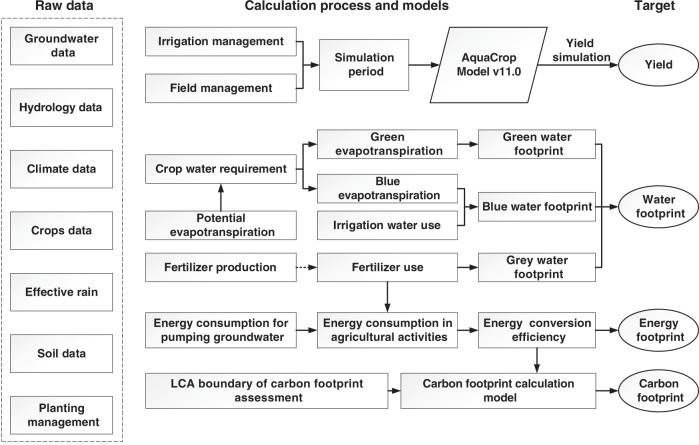
Framework for footprint evaluation model based on LCA. Framework of crop production simulation and water, energy, and carbon footprint assessment in the North China Plain.

We developed and followed the framework (Fig. 5) to figure out environmental impacts of crop production in the NCP (see details in “Methods”).

### Impacts on the spillover system

To evaluate the environmental spillover effects of crop production in the NCP on the spillover system, we analyzed data about water loss and land use change in Hubei Province due to the construction of the SNWTP from the general report on the feasibility study of the first phase project of the Middle Route of the SNWTP. We also assessed CO_2_ emissions and energy footprint from the SNWTP’s life cycle (see details in SI). Because ~22.11% of the total water diverted by the SNWTP goes toward agriculture in the NCP, we multiplied the total CO_2_ emissions and energy footprint of the SNWTP by 22.11% to calculate the environmental impacts of the SNWTP due to crop production in the NCP.

### Water footprint, CO_2_ emissions, and energy footprint from the SNWTP

#### Water footprint

The water footprint of the South-to-North Water Transfer Project mainly includes two parts. One is the water footprint in the process of water transfer (*WF*_e_), which mainly indicates the footprint of the evaporating water surface in the channel water period (*WF*_m_), mainly including material water footprint and construction water footprint. The sum of these two water footprints constitutes the total water footprint of the South-to-North Water Transfer Project during its entire life cycle. The calculation formula is as follows:

16 $$WF = WF_{\mathrm{e}} + WF_{\mathrm{m}}$$

The water footprint generated during the water delivery process is the evaporative water footprint:

17 $$WF_{\mathrm{e}} = 10 \cdot E \cdot A\cdot T$$

Where, *E* is the amount of surface water evaporation in open channels (mm); *A* is the surface area of open channels (hm^2^); *T* is the running time of water, 365 days in this study; 10 is the unit conversion. In this study, the channel water surface evaporation model was adopted for the open channel liquid surface evaporation water footprint. It refers to the Penman formula and Dalton model and was perfected on this basis, so as to propose a multi-factor combined water surface evaporation calculation model. Water surface evaporation calculation model:

18 $$\begin{array}{c}E = {\Delta} e \times f({\Delta} T,r,W)\\ f\left( {{\Delta} T,r,W} \right) = g\left( T \right)\cdot \varphi \left( r \right)\cdot \varphi (W)\\ \varphi \left( W \right) = \left\{ {\begin{array}{*{20}{c}} {0.192 + 0.08W,} & {W \le 1.5\,{\mathrm{m}}{\mathrm{/}}{\mathrm{s}}} \\ {0.312 + 0.078(W - 1.5)^{1 - 0.098(W - 1.5)^{0.5}},} & {W \, > \, 1.5\,{\mathrm{m}}{\mathrm{/}}{\mathrm{s}}} \end{array}} \right.\\ \varphi \left( r \right) = 0.153 + 0.651(1 - r^2)^{1/2}\\ g\left( {{\Delta} T} \right) = 0.92 + 0.0363{\Delta} T^{1.08}\\ {\Delta} e = E_{\mathrm{a}} - E_{\mathrm{w}}\\ E_{\mathrm{a}} = 0.611 \times e^{\frac{{17.27 \times T_{\mathrm{a}}}}{{T_{\mathrm{a}} + 237.2}} \times (1 - \frac{r}{{100}})}\\ E_{\mathrm{w}} = 0.6018 \, \times e^{\frac{{17.25 \times T_{\mathrm{s}}}}{{T_{\mathrm{s}} + 237.3}}}\end{array}$$

where *E* is the evaporation of the reservoir water surface (m^3^); Δ*e* is the difference in saturation vapor pressure; *g* (Δ*T*) is the water temperature difference function; *φ*(*r*) is the relative humidity function; *φ*(*W*) is the wind speed function; *T* is the difference between water temperature and air temperature (°C); *r* is relative humidity (%); *W* is wind speed (m/s); *E*_a_ is the saturated vapor pressure under air (kPa); *E*_w_ is the saturated vapor pressure of water (kPa)); *T*_a_ is the temperature (°C); *T*_s_ is the wet bulb temperature (°C).

For a water delivery channel, its cross-section (including channel bottom width and slope angle) and specific drop have been fixed after completion, while the width of the surface varies with the diversion flow and flow rate. Since the water transfer volume of the South-to-North Water Transfer Project is gradually increasing, the liquid surface area of the water transfer will change.

19 $$F = \sqrt {d^2 + 4\frac{Q}{v}ctg\alpha } \cdot L$$

where, *F* is the open channel water surface area (m^2^); *d* is the bottom width (m); *Q* is the open channel flow (m^3^/s); *v* is the flow rate (m/s); *α* is the open channel slope coefficient; and *L* is the open channel length (M).

The water footprint of energy consumption materials in the life cycle, the production and processing of raw materials in the construction of water transmission channels must be accompanied by the transfer and consumption of water resources, such as steel and concrete. The construction stage of the water conveyance channel includes various construction techniques such as earth and stone excavation and concrete mixing and pouring. Each single project is completed by cooperation of multiple machines. During this process, the power consumption of the construction machinery also produces a certain amount of water footprint. At the same time, concrete curing and mortar mixing are accompanied by a lot of water consumption.

### CO_2_ emissions and energy footprint from the SNWTP

We applied a hybrid EIO-LCA method to understand the CO_2_ emissions of the South-to-North Water Transfer Project (SNWTP)^84^. The system being studied consists of material manufacturing, transportation of material, project construction, and project operation. The disposal of the SNWTP is not included in this study because it is very hard to imagine how such a large project would be disposed. One ton of water transferred per second is considered as the functional unit since the function of the SNWTP is transferring water.

In the material manufacturing process, some materials like cement, steel, wood, and diesel are made. The CO_2_ emissions/energy footprint from material manufacturing is calculated by Eq. (20):

20 $$CF_m = \mathop {\sum}\limits_i {\beta _i \times C_i}$$

where *CF*_*m*_ is the carbon/energy footprint of material manufacturing; *β*_*i*_ represents the CO_2_ emissions/energy consumption intensity factor of material *i*; *C*_*i*_ is the amount of *i* material consumption.

Since the manufacturing sites are distributed widely throughout China, we derived the average distance of different kinds of materials transported from the manufacturing sites to the construction site from China Statistical Yearbook 2002, and then calculated diesel or gasoline consumed during the transportation stage. By hydraulic quota 2002, we chose 45 t freight truck as the distant transportation tool to transport different kinds of materials. Carbon/energy footprint from transportation stage *CF*_t_ can be calculated as follows:

21 $$CF_{\mathrm{t}} = \mathop {\sum}\limits_i {\beta _{\mathrm{d}}\alpha _{\mathrm{d}} \times \frac{{Q_i}}{L} \times \frac{{s_i}}{v}}$$

where *β*_d_ represents CO_2_ emissions/energy consumption intensity factor for diesel fuel; *α*_d_ represents the amount of diesel consumption by truck per hour; *Q*_*i*_ is the quantities of material transported; *L* represents the load of truck. *s*_*i*_ represents the distance of transportation; *v* is the speed of the truck.

Within the construction process, various work activities are involved, such as concrete mixing, concrete lining, soil-rock excavation, and soil-rock filling. The CO_2_ emissions are produced by diesel and electricity consumed during construction, which can be calculated by using Eqs. (22)–(24):

22 $$C_{{\mathrm{d}}i} = \frac{{w_i}}{{h_i}} \times k_i$$

23 $$C_{{\mathrm{e}}i} = \frac{{w_i}}{{h_i}} \times g_i$$

24 $$CF_{\mathrm{c}} = \mathop {\sum}\limits_i {(\beta _{\mathrm{d}}C_{{\mathrm{d}}i} + \beta _{\mathrm{e}}C_{{\mathrm{e}}i}).}$$

where *CF*_c_ represents the total carbon/energy footprint from construction; *C*_d__*i*_ means the diesel consumption of work *i*; *C*_e__*i*_ is the electricity consumption in work *i*; *β*_e_ represents the CO_2_ emissions/energy coumption intensity factor of electricity, *β*_d_ is the CO_2_ emissions/energy consumption intensity factor of diesel; *w*_*i*_ is the total amount of work *i*, and *h*_*i*_ represents the amount of work finished per unit time; *k*_*i*_ is the diesel consumed per unit time, and *g*_*i*_ represents the electricity consumed per unit time.

We applied economic input-output (EIO) life cycle assessment to quantify carbon/energy footprint from operation stage. According to a survey^85^, for concrete construction, each year the maintenance stage would cost 2% of total expense of one project, thus we also set this ratio for the SNWTP. Based on the standard for the “*Economic Evaluation of Water Conservancy Construction Project (SL72-2013)*”^42^, the SNWTP’s life of maintenance and operation spans 50 years^86^. After obtaining the total cost of the maintenance and operation stage, we used the EIO method to derive the CO_2_ emissions from maintenance and operation stage by choosing construction, nonresidential maintenance and repair sector in the US 2002 Benchmark EIO model and running it.

### Data quality assessment methods and models

Life cycle assessment (LCA) is an evaluation method based on the calculation and analysis of the data list in the entire life cycle. The uncertainty of the original data and the calculation process method affect the accuracy of the life cycle assessment results to some extent. Therefore, it is often necessary to analyze the uncertainty of the results from LCA. In this study, an evaluation method combining data quality index method (DQI) and uncertainty was used to establish a data quality evaluation model, and the final results were simulated through Monte Carlo Simulation to obtain the uncertainty of outcomes.

### Step 1: scoring data quality

This study used a data quality scoring system based on five perspectives: credibility, completeness, technology-related, time-related, and geographic-related. The data quality was divided into five levels according to the data quality index matrix and assignment criteria. The larger the score, the better the quality of the data. By analyzing the data list, a data quality vector was formed, and the weighted average was calculated as:

25 $$\bar q = \mathop {\sum}\limits_{i = 1}^n {q_i}$$

where $\bar q$ is the data quality of the original data; *n* (1–5) is the index of the vector with 5 elements (i.e., five data perspectives); *q*_*i*_ is the data quality vector element value.

### Step 2: the range of the indicator *R* is calculated as

26 $$R = \frac{{\bar q - {\mathrm{min}}q_i}}{{{\mathrm{max}}q_i - {\mathrm{min}}q_i}} \times 100{\mathrm{\% }}$$

where *R* is the percentage of $\bar q$ within the total quality range of the data quality vector; min*q*_*i*_ is the minimum vector element value, and max*q*_*i*_ is the maximum vector element value. According to *R*, the data quality vector was converted into the data quality indicators (DQI), and the data are shown in Supplementary Table 4.

### Step 3: input data probability distribution

After determining DQI, it is necessary to determine the probability distribution of the original data and fit the data to it. This study used a wide range of *β* distribution density functions, the formula was as follows:

27 $$f\left( {x;\alpha ;\beta ;a;b} \right) =	 \left[ {\frac{1}{{b - a}}} \right] \times \left\{ {{\Gamma} (\alpha + \beta ){\mathrm{/}}\left[ {{\Gamma} (\alpha ) \times {\Gamma} (\beta )} \right]} \right\} \times \left[ {(x - a){\mathrm{/}}(x - b)} \right]^{\alpha - 1} \\ 	\times \left[ {\frac{{b - x}}{{b - a}}} \right]^{\beta - 1}$$

where *α* and *β* are distribution shape parameters, *a* and *b* are selected range breakpoints.

### Step 4: uncertainty calculation

The original data distribution type is determined by comparing the *β* random distribution parameter table of the data quality index DQI (Supplementary Table 4). The uncertainty *U*_0_ of the original data can be expressed by the relative standard deviation (RSD) of the distribution type. In this study, original data with *U*_0_ < 10% (i.e., the uncertainty of the original data is less than 10%) was considered to be of good quality^87^.

### Step 5: Monte Carlo simulation

Monte Carlo simulations with 50,000 times and a 95% confidence interval were carried out to obtain the probability distribution of data from each stage and obtain the uncertainty of the final LCA product (Supplementary Fig. 8)^87^.

Because the calculation of the footprint using LCA includes different stages with the use of data from different sources at different stages, there are several uncertainties associated with data collection at each stage, it is hard to account for all possible data uncertainties in LCA. Hence, the accuracies of LCA results are highly prone to errors arose from these uncertainties. This study used the data quality evaluation standard to evaluate the quality of data for agricultural production and the parameters during each stage of the South-to-North Water Transfer Project based on each element. Scoring was performed based on a DQI-based scoring system. Based on the original data quality matrix, the footprint results of each stage were simulated to determine the distribution type, and the uncertainty *U*_0_ of the original data under this distribution type was obtained. The data uncertainty from each stage was found to be between 3 and 6%, well below the 10% threshold. Hence, the data used in this study were reliable^88^.

### Reporting summary

Further information on research design is available in the Nature Research Reporting Summary linked to this article.

## Supplementary information

Supplementary Information

Reporting Summary

## Data Availability

All data generated during this study are available from the corresponding author upon reasonable request. Source data are provided with this paper.

## Source data

Source Data

## Code availability

All computer codes generated during this study are available from the corresponding author upon reasonable request.
