# Supplementary material for: Impacts of irrigated agriculture on food–energy–water–CO2 nexus across metacoupled systems
Source: Nat Commun. 2020 Nov 17;11:5837. doi: 10.1038/s41467-020-19520-3 (PMC7672069; doi:10.1038/s41467-020-19520-3)
Supplement: Supplementary file 1 — Supplementary Information [file 41467_2020_19520_MOESM1_ESM.pdf]

Supplementary Information

Impacts of irrigated agriculture on food-energy-water-CO_2 nexus across metacoupled systems

By Xu[1,2] et al

[1]Center for Systems Integration and Sustainability, Department of Fisheries and Wildlife, Michigan State University, East Lansing 48823, USA.

[2] School for Environment and Sustainability, University of Michigan, Ann Arbor 48109, USA.

Supplementary Table and Figures

Supplementary Table 1. Food yield, energy-water-carbon footprints, water and food sustainability under different scenarios

Name	Yield (t)	Water Footprint (m^3)	Energy footprint (MJ)	Carbon footprint (t)	Water sustainability	Food sustainability
Baseline	5.46×10^{7}	1.78×10^{10}	4.81×10^{12}	6.55×10^{7}	0.23	1.10
S1	5.47×10^{7}	1.78×10^{10}	4.81×10^{12}	6.55×10^{7}	0.23	1.10
S2	5.02×10^{7}	1.75×10^{10}	4.57×10^{12}	6.47×10^{7}	0.23	1.01
S3	4.85×10^{7}	1.73×10^{10}	4.43×10^{12}	6.40×10^{7}	0.24	0.98
S4	4.91×10^{7}	1.75×10^{10}	4.57×10^{12}	6.47×10^{7}	0.23	0.99
S5	6.77×10^{7}	0.89×10^{10}	4.76×10^{12}	6.59×10^{7}	0.46	1.36
S6	6.52×10^{7}	1.29×10^{10}	4.98×10^{12}	6.81×10^{7}	0.32	1.31
S7	4.15×10^{7}	1.17×10^{10}	3.06×10^{12}	5.77×10^{7}	0.35	0.84
S8	5.60×10^{7}	0.59×10^{10}	3.19×10^{12}	5.80×10^{7}	0.69	1.13
S9	5.40×10^{7}	0.86×10^{10}	3.34×10^{12}	5.95×10^{7}	0.48	1.09
S10	4.19×10^{7}	0.67×10^{10}	2.60×10^{12}	5.56×10^{7}	0.61	0.84
S11	2.58×10^{7}	0.38×10^{10}	1.50×10^{12}	4.98×10^{7}	1.08	0.52
S12	2.58×10^{7}	0.17×10^{10}	7.07×10^{11}	4.56×10^{7}	2.42	0.52
S13	5.40×10^{7}	0.86×10^{10}	3.34×10^{12}	6.26×10^{7}	0.75	1.09
S14	5.47×10^{7}	0.86×10^{10}	3.35×10^{12}	6.58×10^{7}	2.75	1.10
S15	5.47×10^{7}	0.17×10^{10}	7.17×10^{11}	5.20×10^{7}	3.40	1.10

Supplementary Table 2. Water- and energy-saving potential under different scenarios

Name	Water-saving potential (m^3)	Energy-saving potential (MJ)
S1	0	0
S2	3.0×10^{8}	8.0×10^{5}
S3	5.0×10^{8}	1.5×10^{6}
S4	3.0×10^{8}	8.0×10^{5}
S5	8.9×10^{9}	-4.0×10^{5}
S6	4.9×10^{9}	-2.6×10^{5}
S7	6.1×10^{9}	7.8×10^{6}
S8	1.2×10^{10}	7.5×10^{6}
S9	9.2×10^{9}	6.0×10^{6}
S10	1.1×10^{10}	9.9×10^{6}
S11	1.4×10^{10}	1.6×10^{7}
S12	1.6×10^{10}	2.0×10^{7}
S13	9.2×10^{9}	2.9×10^{6}
S14	9.2×10^{9}	-3.0×10^{5}
S15	1.6×10^{10}	1.4×10^{7}

Supplementary Table 3. Irrigation technologies and efficiency factors [1-4]

Technology Upgrade to	Water saving rate (%)	Fertilizer saving rate (%)	Yield increasing rate (%)	Carbon footprint (kg hm^{-2} y^{-1})
Drip irrigation	60-85	30-50	10-15	+12.8
Sprinkler irrigation	50-75	20-40	15-20	+13.4

Supplementary Table 4. Data Quality Index (DQI)

Percentage of quality data	Data Quality Index (DQI)
0≤R＜12.5%	1.0
12.5%≤R＜25%	1.5
25%≤R＜37.5%	2.0
37.5%≤R＜50%	2.5
50%≤R＜62.5%	3.0
62.5%≤R＜75%	3.5
75%≤R＜87.5%	4.0
87.5%≤R＜100%	4.5
R=100%	5.0

Supplementary Table 5. Distribution shape parameters

DQI	β distribution parameter (α，β)	Range endpoint (+/-, %)
5.0	（5，5）	10
4.5	（4，4）	15
4.0	（3，3）	20
3.5	（2，2）	25
3.0	（1，1）	30
2.5	（1，1）	35
2.0	（1，1）	40
1.5	（1，1）	45
1.0	（1，1）	50

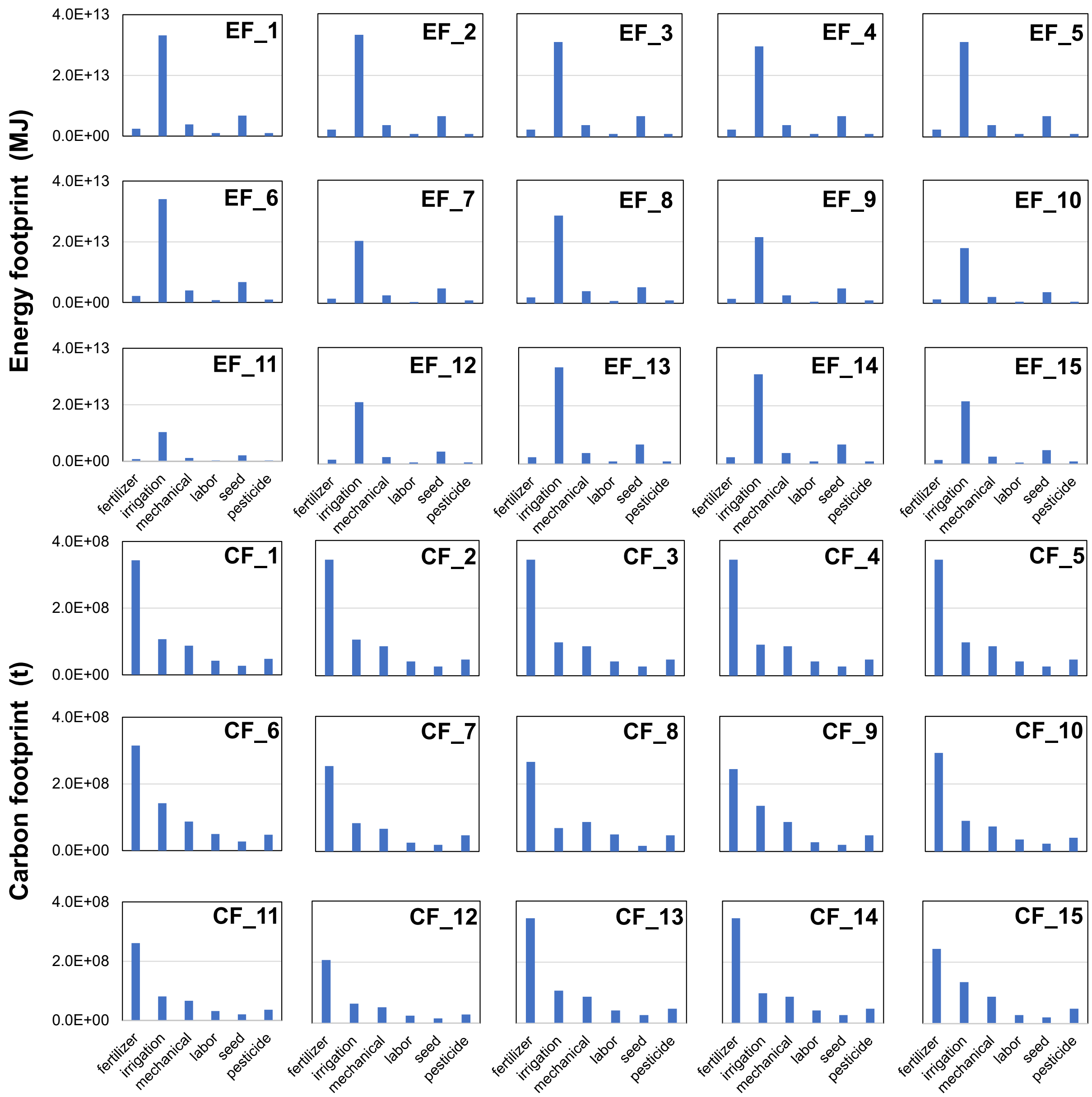

Supplementary Fig. 1 Energy and carbon footprints at different stages of the life cycle in irrigated agriculture of the NCP under various scenarios.

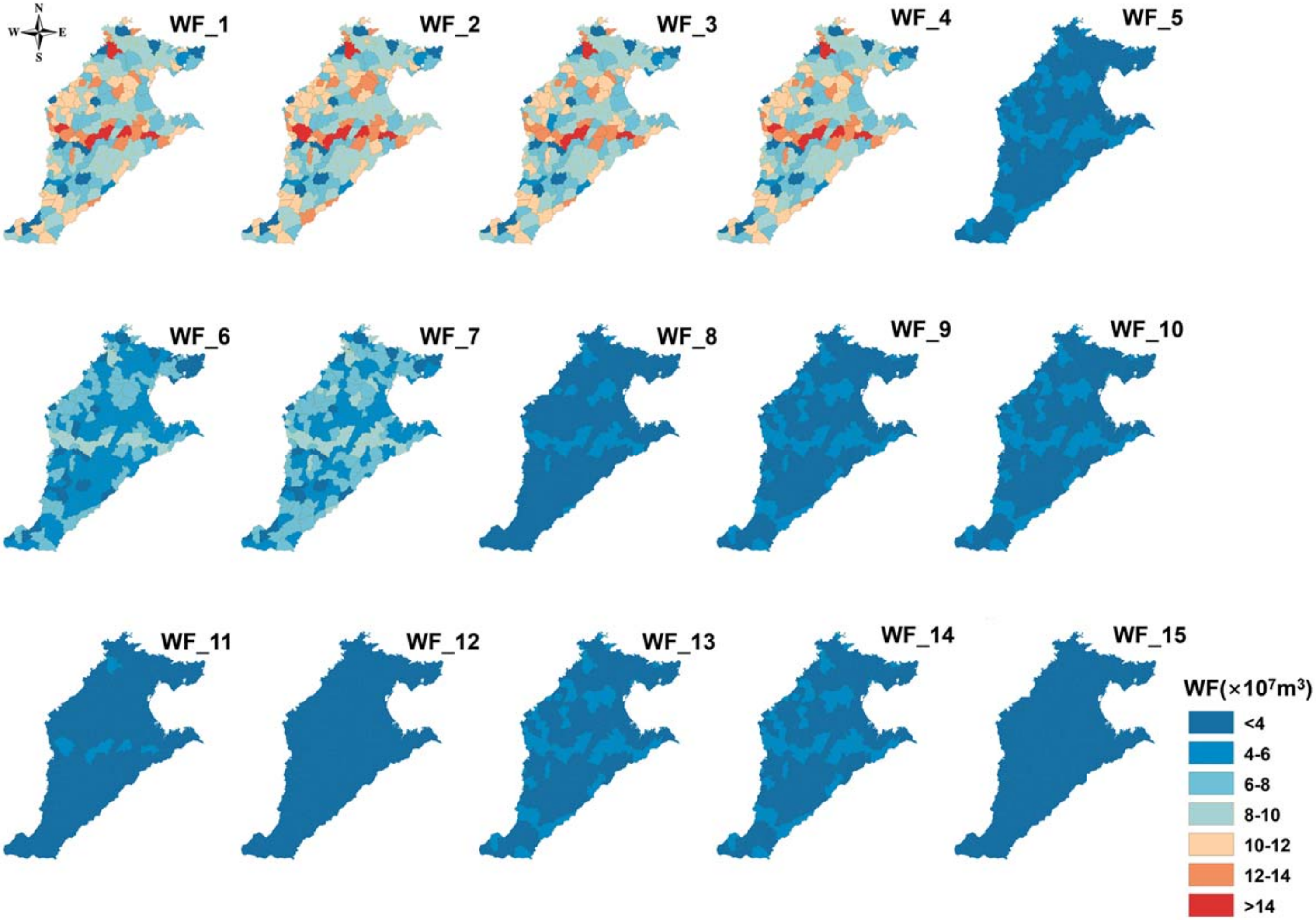

Supplementary Fig. 2 Spatial dynamics of water footprint (WF_1-15) in irrigated agriculture of the NCP under scenarios S1-S15. The data for the base map were derived from the Resource and Environment Science and Data Center (http://www.resdc.cn/) which is public available. The boundary of the North China Plain was created by the authors (using ArcGIS version 10.1, ESRI).

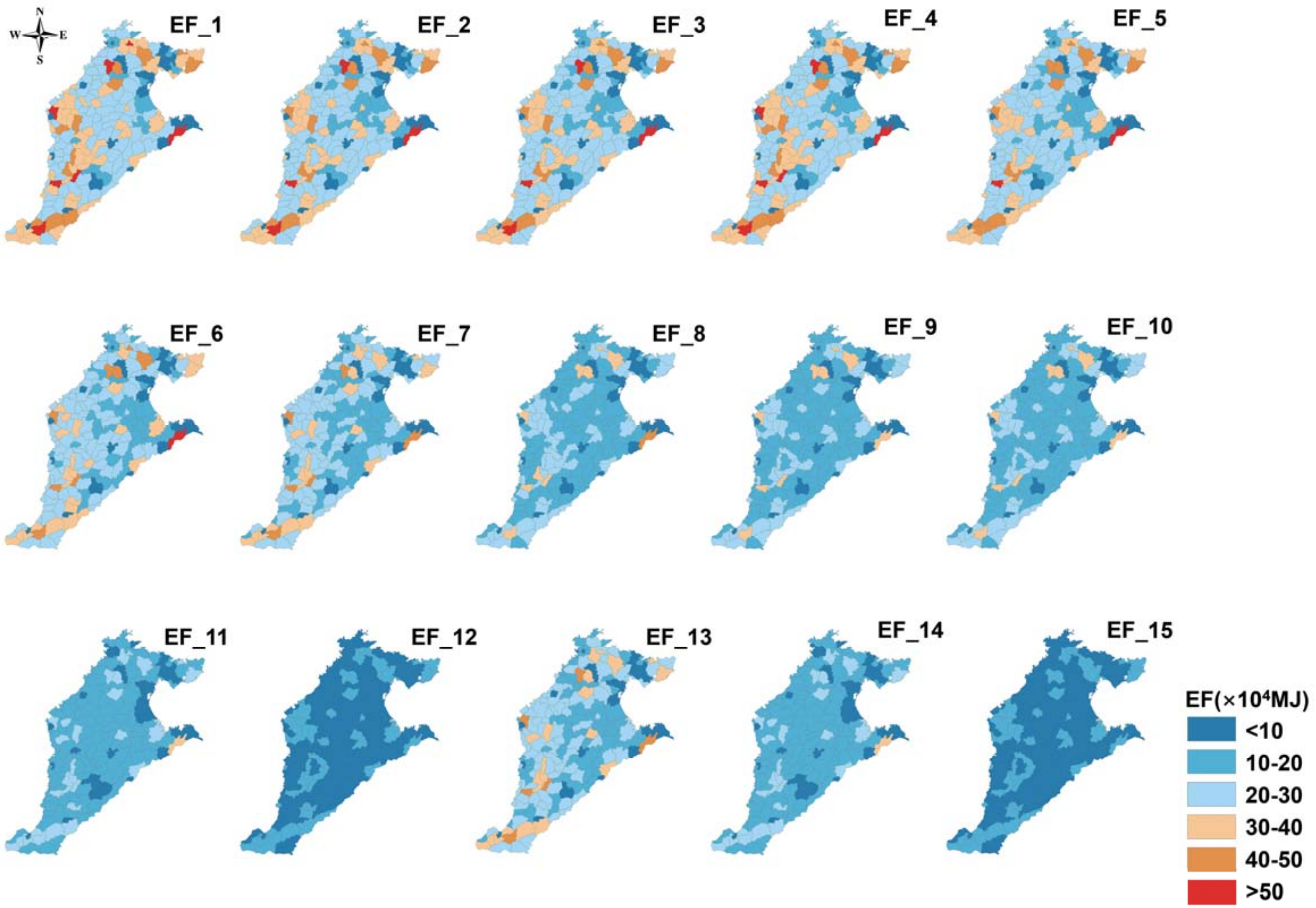

Supplementary Fig. 3 Spatial dynamics of energy footprint (EF_1-15) in irrigated agriculture of the NCP under scenarios S1-S15. The data for the base map were derived from the Resource and Environment Science and Data Center (http://www.resdc.cn/) which is public available. The boundary of the North China Plain was created by the authors (using ArcGIS version 10.1, ESRI).

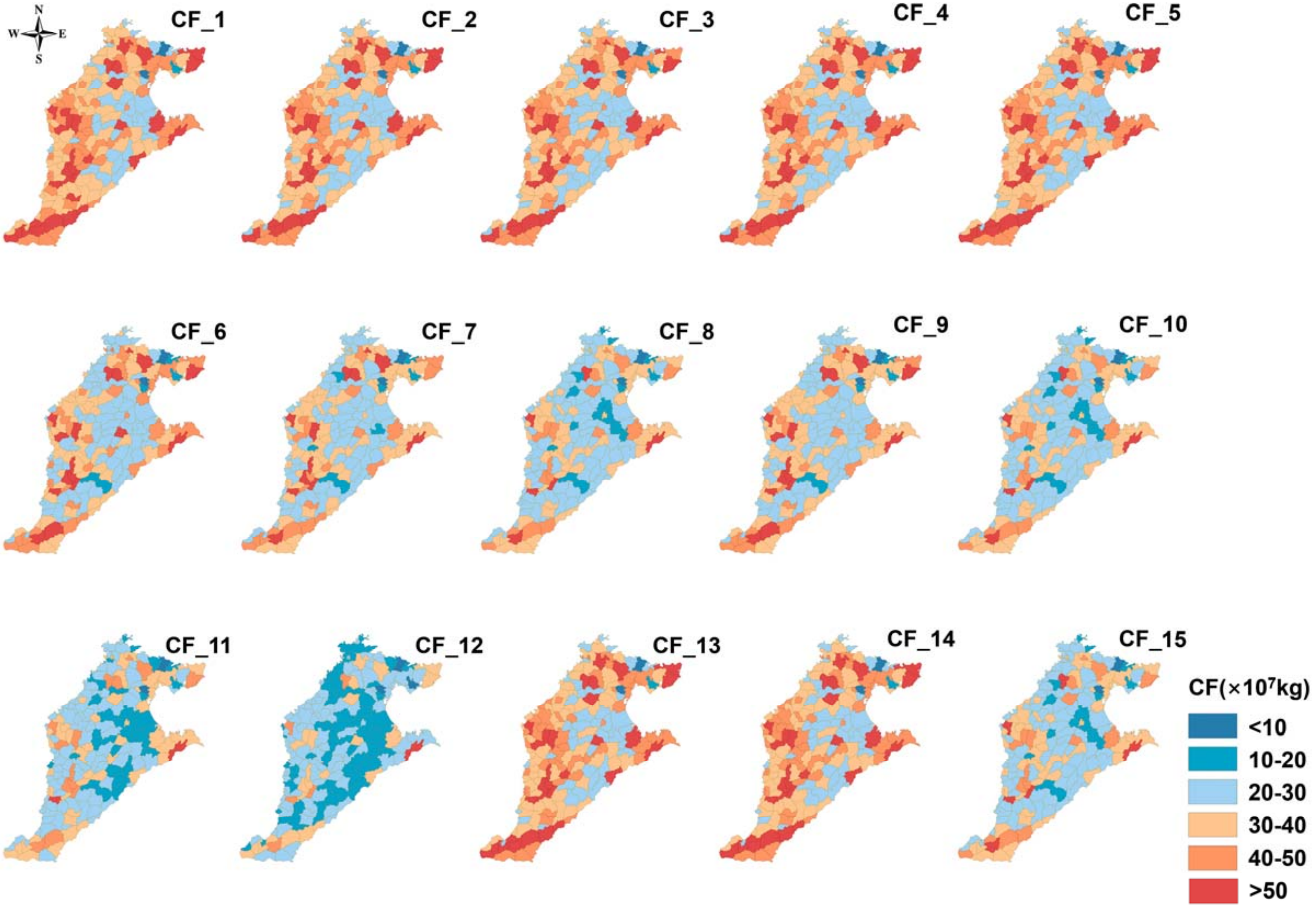

Supplementary Fig. 4 Spatial dynamics of carbon footprint (CF_1-15) in irrigated agriculture of the NCP under scenarios S1-S15. The data for the base map were derived from the Resource and Environment Science and Data Center (http://www.resdc.cn/) which is public available. The boundary of the North China Plain was created by the authors (using ArcGIS version 10.1, ESRI).

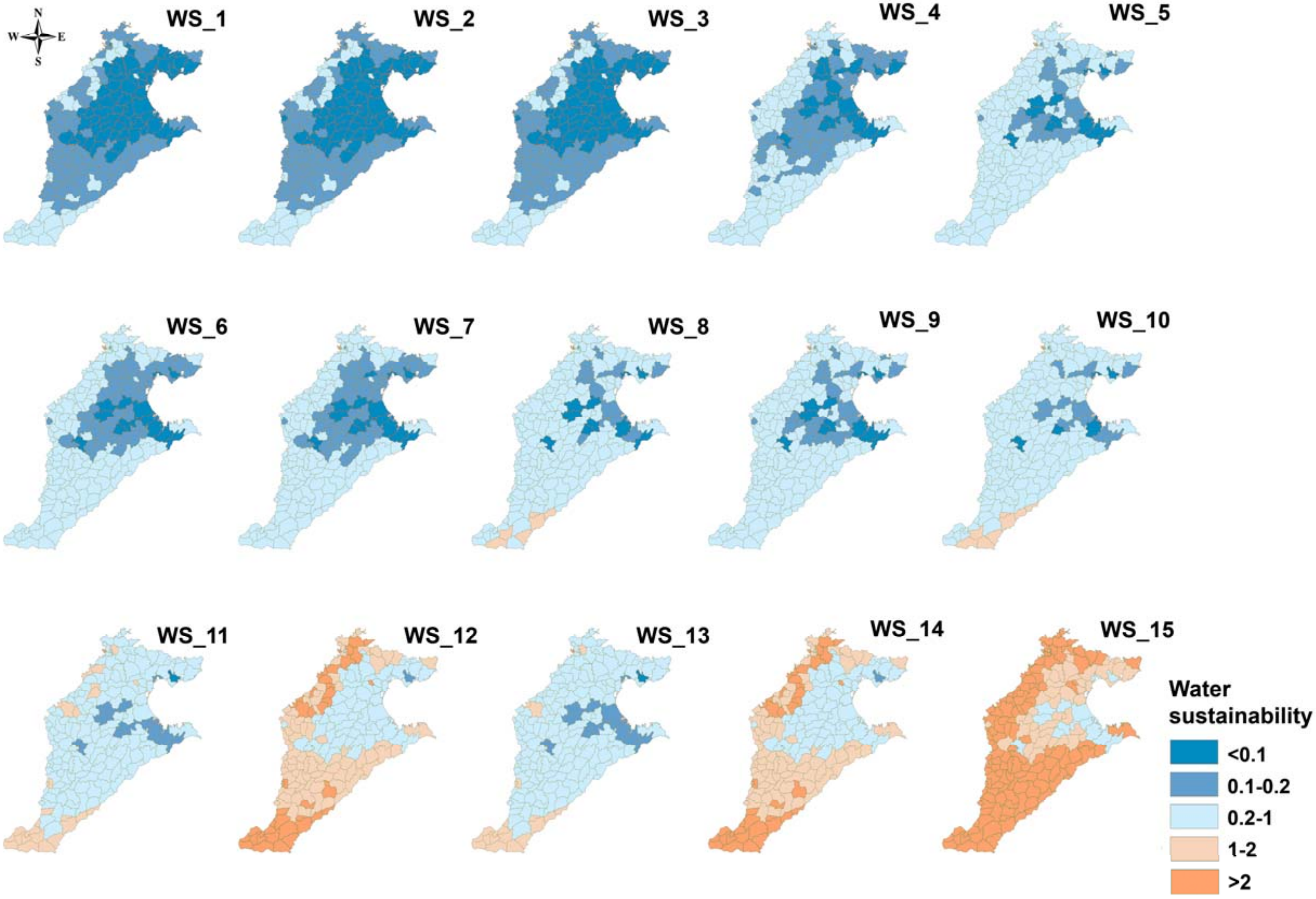

Supplementary Fig. 5 Spatial dynamics of water sustainability (WS_1-15) in irrigated agriculture of the NCP under scenarios S1-S15. The data for the base map were derived from the Resource and Environment Science and Data Center (http://www.resdc.cn/) which is public available. The boundary of the North China Plain was created by the authors (using ArcGIS version 10.1, ESRI).

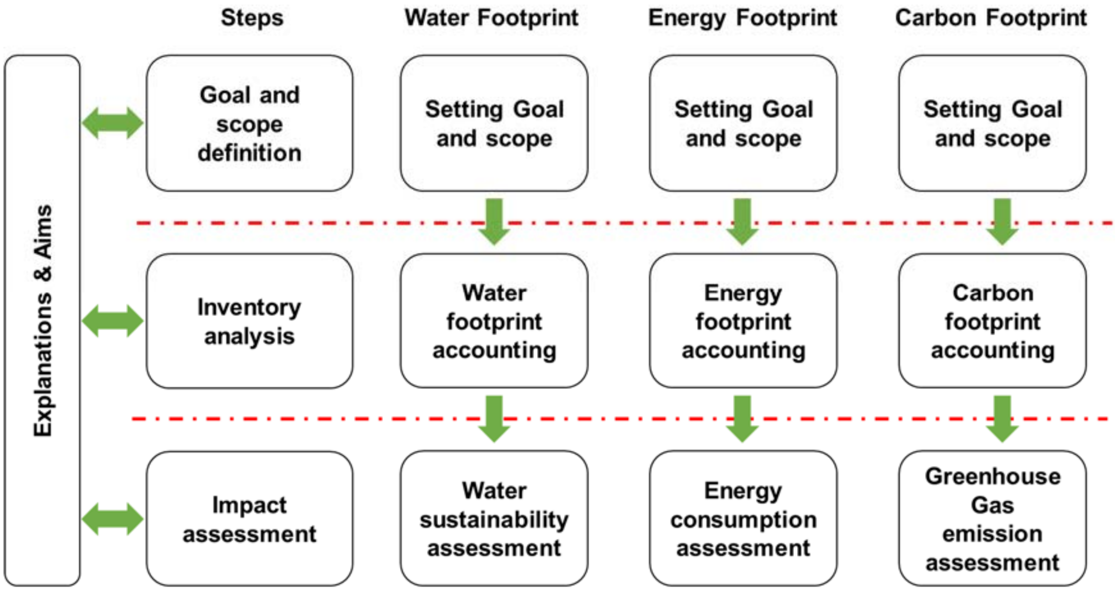

Supplementary Fig. 6 Integrated framework of LCA and footprints assessment

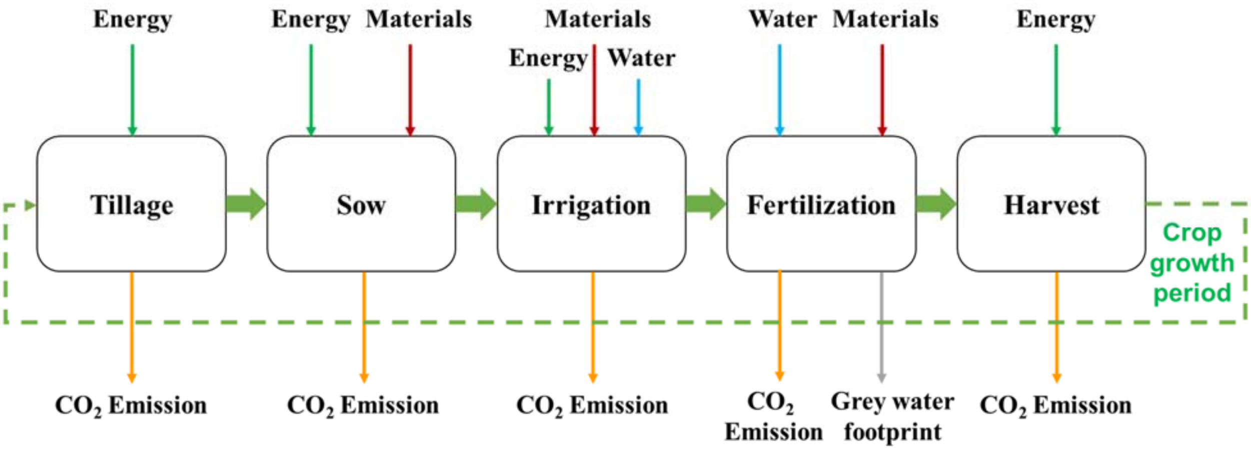

Supplementary Fig. 7 System phases, boundaries and functional unit

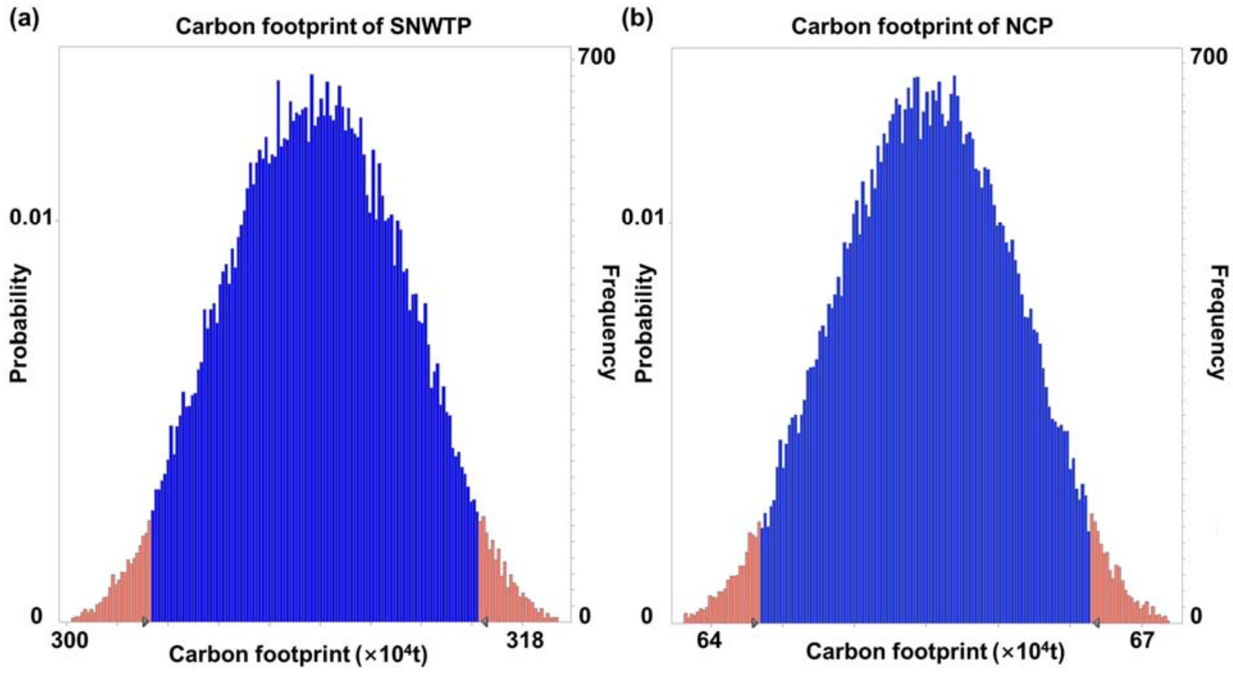

Supplementary Fig. 8 Monte Carlo Simulations of carbon footprint. The probability distribution of carbon footprints from Monte Carlo simulation (50,000 simulations) showing a relatively normal distribution, indicating that the calculation model is reliable and all are in line with the actual situation, which presents grounds for the reliability of the results from this study.

Supplementary References

1 Ziogou, I., Michopoulos, A., Voulgari, V. & Zachariadis, T. Energy, environmental and economic assessment of electricity savings from the operation of green roofs in urban office buildings of a warm Mediterranean region. *Journal of cleaner production* **168**, 346-356 (2017).

2 Chen, X. *et al.* Environmental impact assessment of water-saving irrigation systems across 60 irrigation construction projects in northern China. *Journal of Cleaner Production* **245**, 118883 (2020).

3 Sinha, I., Buttar, G. & Brar, A. Drip irrigation and fertigation improve economics, water and energy productivity of spring sunflower (Helianthus annuus L.) in Indian Punjab. *Agricultural Water Management* **185**, 58-64 (2017).

4 Kandelous, M. M. *et al.* Evaluation of subsurface drip irrigation design and management parameters for alfalfa. *Agricultural Water Management* **109**, 81-93 (2012).
