# Supplementary material for: Impacts of irrigated agriculture on food–energy–water–CO2 nexus across metacoupled systems
Source: Nat Commun. 2020 Nov 17;11:5837. doi: 10.1038/s41467-020-19520-3 (PMC7672069; doi:10.1038/s41467-020-19520-3)
Supplement: Supplementary file 2 — Reporting Summary [file 41467_2020_19520_MOESM2_ESM.pdf]

nature research

Corresponding author(s): Jianguo (Jack) Liu

Last updated by author(s): 08/06/2020

Reporting Summary

Nature Research wishes to improve the reproducibility of the work that we publish. This form provides structure for consistency and transparency in reporting. For further information on Nature Research policies, see our Editorial Policies and the Editorial Policy Checklist.

Statistics

For all statistical analyses, confirm that the following items are present in the figure legend, table legend, main text, or Methods section.

n/a	Confirmed	
☒	☐	The exact sample size (n) for each experimental group/condition, given as a discrete number and unit of measurement
☒	☐	A statement on whether measurements were taken from distinct samples or whether the same sample was measured repeatedly
☒	☐	The statistical test(s) used AND whether they are one- or two-sided<br>*Only common tests should be described solely by name; describe more complex techniques in the Methods section.*
☒	☐	A description of all covariates tested
☒	☐	A description of any assumptions or corrections, such as tests of normality and adjustment for multiple comparisons
☒	☐	A full description of the statistical parameters including central tendency (e.g. means) or other basic estimates (e.g. regression coefficient) AND variation (e.g. standard deviation) or associated estimates of uncertainty (e.g. confidence intervals)
☒	☐	For null hypothesis testing, the test statistic (e.g. F, t, r) with confidence intervals, effect sizes, degrees of freedom and P value noted<br>*Give P values as exact values whenever suitable.*
☒	☐	For Bayesian analysis, information on the choice of priors and Markov chain Monte Carlo settings
☒	☐	For hierarchical and complex designs, identification of the appropriate level for tests and full reporting of outcomes
☒	☐	Estimates of effect sizes (e.g. Cohen's d, Pearson's r), indicating how they were calculated

Our web collection on statistics for biologists contains articles on many of the points above.

Software and code

Policy information about availability of computer code

Data collection	We obtained agrometeorological data from 2000 to 2015 from the Meteorological Data Sharing Service System of National Meteorological Information Center of China, and basic agricultural data (e.g., cultivated area, nitrogen use, winter wheat and summer maize production, and areal extent of other crops) in the North China Plain (NCP) at the county level from the Agricultural Information Institute of Chinese Academy of Agricultural Sciences. We obtained crop evapotranspiration measurements for summer maize and winter wheat from Luancheng Agro-Eco-Experimental Station of the Chinese Academy of Sciences. We received data about South-North Water Transfer Project (SNWTP)'s construction materials, work items and quantity of work from the general report on the feasibility study of the first phase project of the middle route of the SNWTP. Because the construction of the western route has not started, this study focused on the middle route and eastern route. The carbon emission factor and energy intensity factor of each material used for construction were derived from ELCD, IPCC (Intergovernmental Panel on Climate Change) and Ecoinvent databases. According to the general report of SNWTP, all construction processes, such as selecting machines and determining machines' work hours, followed the Hydraulic Construction Mechanical Quota 2002. The hydraulic quota 2002 was used to define the energy consumption of each machine team of the work process.
Data analysis	We use Microsoft Excel 2016, R software and Oracle crystal ball to analyze data.

For manuscripts utilizing custom algorithms or software that are central to the research but not yet described in published literature, software must be made available to editors and reviewers. We strongly encourage code deposition in a community repository (e.g. GitHub). See the Nature Research guidelines for submitting code & software for further information.

Data

Policy information about availability of data

All manuscripts must include a data availability statement. This statement should provide the following information, where applicable:

- Accession codes, unique identifiers, or web links for publicly available datasets
- A list of figures that have associated raw data
- A description of any restrictions on data availability

All data and codes are available from the corresponding author upon reasonable request.

Field-specific reporting

Please select the one below that is the best fit for your research. If you are not sure, read the appropriate sections before making your selection.

☐ Life sciences ☐ Behavioural & social sciences ☒ Ecological, evolutionary & environmental sciences

For a reference copy of the document with all sections, see nature.com/documents/nr-reporting-summary-flat.pdf

Ecological, evolutionary & environmental sciences study design

All studies must disclose on these points even when the disclosure is negative.

Study description	Irrigated agriculture has important implications for achieving the United Nations Sustainable Development Goals (SDGs), especially those related to hunger, water, energy, and carbon emissions. Irrigated agriculture in North China Plain (NCP), China's agricultural base, largely influences China's food security and related resource consumption and environmental sustainability. However, there is a lack of systematic and quantitative analyses on how irrigated agriculture in NCP impacts food, water, energy and carbon emissions simultaneously in China under complex environmental and socioeconomic factors (e.g., climate change, diet change, irrigation technologies, crop planting strategies, water diversion project) even though they are interconnected. To fill this important knowledge gap, we performed a quantitative analysis of environmental impacts associated with food sustainability in China across food sending systems (the North China Plain (NCP), the national agricultural base), food receiving systems (most of the rest of China) and spillover systems (Hubei Province; areas that are affected by interactions between sending and receiving systems under the framework of metacoupling (environmental and socioeconomic interactions within and across borders)). We find that although food supply promotes food sustainability in the rest of China, the NCP consumes over seven times more water than its total annual renewable water, which has led to unsustainable water use in all 207 counties of the NCP. Although Hubei Province was seldom involved in the food trade it experienced substantial losses in water and land due to the construction of the South-to-North Water Transfer Project (SNWTP) which aims to alleviate water shortages in the NCP. The SNWTP also consumes large amounts of energy to transfer water which lead to large carbon emissions. We also found strong variations in water footprint, energy footprint, carbon footprint, water sustainability and crop yield in NCP under various factors (e.g., climate change, diet change, irrigation technologies, crop planting strategies). Only in scenarios S14 and S15 both water sustainability and food sustainability can be achieved, while in other scenarios (S1-S13) at least one type of sustainability cannot be achieved. This study uncovers the relationship between food sustainability and related environmental impacts across sending, receiving, and spillover systems. It emphasizes the need to identify and understand many potential similar consequences in other regions of the world affected by food production and trade to achieve global sustainability.
Research sample	We chose all 207 counties in North China Plain as the sample.
Sampling strategy	We chose all 207 counties in North China Plain as the sample.
Data collection	We obtained agrometeorological data from 2000 to 2015 from the Meteorological Data Sharing Service System of National Meteorological Information Center of China, and basic agricultural data (e.g., cultivated area, nitrogen use, winter wheat and summer maize production, and areal extent of other crops) in the NCP at the county level from the Agricultural Information Institute of Chinese Academy of Agricultural Sciences. We obtained crop evapotranspiration measurements for summer maize and winter wheat from Luancheng Agro-Eco-Experimental Station of the Chinese Academy of Sciences. We obtained data about SNWTP's construction materials, work items and quantity of work from the general report on the feasibility study of the first phase of the middle route of the SNWTP. Because construction of the western route has not started yet, this study focused on the middle route and eastern route. The carbon emission factor and energy intensity factor of each material used for construction were derived from ELCD, IPCC (Intergovernmental Panel on Climate Change) and Ecoinvent databases. According to the general report of SNWTP, all construction processes, such as selecting machines and determining machines' work hours, followed the Hydraulic Construction Mechanical Quota 2002. The hydraulic quota 2002 was used to define the energy consumption of each machine team of the work process.
Timing and spatial scale	We studied NCP and China from 2000 to 2020.
Data exclusions	All data were included
Reproducibility	We have provided data source files to ensure reproducibility
Randomization	All counties in NCP were classified as one group

Blinding	No blinding.

Did the study involve field work? ☐ Yes ☒ No

Reporting for specific materials, systems and methods

We require information from authors about some types of materials, experimental systems and methods used in many studies. Here, indicate whether each material, system or method listed is relevant to your study. If you are not sure if a list item applies to your research, read the appropriate section before selecting a response.

Materials & experimental systems

n/a	Involved in the study
☒	☐ Antibodies
☒	☐ Eukaryotic cell lines
☒	☐ Palaeontology and archaeology
☒	☐ Animals and other organisms
☒	☐ Human research participants
☒	☐ Clinical data
☒	☐ Dual use research of concern

Methods

n/a	Involved in the study
☒	☐ ChIP-seq
☒	☐ Flow cytometry
☒	☐ MRI-based neuroimaging
